# Changing epidemiology of yellow fever virus in Oyo State, Nigeria

**DOI:** 10.1186/s12889-022-12871-0

**Published:** 2022-03-08

**Authors:** Bassey Enya Bassey, Fiona Braka, Rosemary Onyibe, Olufunmilola Olawumi Kolude, Marcus Oluwadare, Alawale Oluwabukola, Ogunlaja Omotunde, Oluwatobi Adeoluwa Iyanda, Adedamola Ayodeji Tella, Olayiwola Suliat Olanike

**Affiliations:** World Health Organization (WHO) Nigeria Country Office, UN House, Plot 617/618, Diplomatic Drive, Central Business District, PMB 2861, Garki, Abuja, Nigeria

**Keywords:** Yellow fever, Resurgence, Outbreaks, Surveillance, And immunization, EPI

## Abstract

**Background:**

Yellow Fever is an acute viral hemorrhagic disease endemic in tropical Africa and Latin America and is transmitted through infected mosquitoes. The earliest outbreak of yellow fever in Nigeria was reported in Lagos in 1864 with subsequent regular outbreaks reported until 1996. A large epidemic of yellow fever occurred in Oyo State in April and May 1987 following an epidemic of sylvatic yellow fever in Eastern Nigeria the previous year. For 21 years, no further confirmed cases were reported until September 2017 following which Nigeria has been responding to successive outbreaks. The renewed onset of yellow fever outbreaks in Nigeria followed a global trend of reports and from other African countries. Yellow Fever disease has no cure, but control is through vaccination and vector control. Eliminating Yellow fever Epidemic (EYE) strategy to improve high-risk countries’ prevention, preparedness, detection, management, and response to yellow fever outbreaks was developed by the WHO in 2017 and launched in Nigeria in April 2018. Yet, poor vaccination coverage continues to be a cause for concern.

**Materials and methods:**

We conducted a retrospective cross-sectional study that examines the resurgence of Yellow fever cases and outbreaks from 2013 to 2020 in Oyo State, Nigeria. The Yellow Fever data for both surveillance and routine Expanded Programme on Immunization (EPI) were the focus of the review. Surveillance data were retrieved from the State’s database reported by all 33 LGAs, maintained by the State and supported by the World Health Organization at the Zonal and State levels. The routine EPI data were retrieved from District Health Information Software (DHIS_2). The proportion of LGAs reporting at least one case of suspected yellow fever with a blood specimen and the number of suspected cases reported for each year within the period under review was measured. We also assessed the trend of confirmed cases and the incidence per 100,000 persons. Also, suspected cases of yellow fever were categorized into four age groups and their vaccination status was assessed. The State’s annual administrative routine vaccination coverage for yellow fever vaccine was compared with the number of confirmed cases for each year.

**Results:**

The proportion of LGAs reporting at least a case of suspected yellow fever, with blood samples collected, ranged from 6.1 to 84.9% between 2014 and 2020 while a total of 9 confirmed (8 cases) and probable (1 case) cases of yellow fever were recorded. However, there were no confirmed cases from the year 2013 to 2016, including 2018 but an upward trend of incidence of the disease per 100,000 persons from 0% to 2013 through 2018, to 3.5% in 2019, and then to 5.6% in 2020 was observed. 93 of 240 (39%) suspected yellow fever cases reported during the given period were observed to have received yellow fever vaccine.

**Conclusions:**

In conclusion, the increase in the circulation of the yellow fever virus in the state reiterates the state is at a high risk of yellow fever transmission and underlines the need for viable interventions such as environmental hygiene to rid the environment of the disease vector’s ecological niche and improving routine EPI coverage to provide population immunity.

## Introduction

Yellow Fever is an acute viral hemorrhagic disease caused by a single-stranded RNA virus belonging to the genus *flavivirdae*. It is endemic in tropical Africa as well as Central and South America and transmitted through infected mosquitoes [1]. Aedes sp is mostly responsible for spread in Africa while the *Aedes, Haemagogus*, and *Sabethes spp* are responsible in South America [2]. There are three transmission cycles of the spread of the disease, the Jungle also referred to as Sylvatic, Intermediate, also called Savannah, and the urban transmission cycles. For the Jungle cycle, the vector, *Aedes sp, Haemagogus/ Sabethes spp* mosquitoes in the forest bite monkeys, which are the primary reservoir of the yellow Fever virus and spread the virus. The intermediate transmission cycle, common in Africa, involves infected mosquitoes biting humans that work or live close to forests while for the urban cycle, majorly responsible for sustained outbreaks, the spread is from infected persons to other persons via the same vector [3, 4].

In Africa’s endemic areas, there is increased natural immunity with age, thus infants and children are at the highest risk of infection whereas, in the Americas, most cases reported were unimmunized men believed to be exposed to infected mosquitoes while working in forested areas [2].

The global burden of Yellow Fever is estimated at 200,000 cases and 30,000 deaths annually with case fatality that may be as high as 50% in untreated severely affected [5] and Africa is said to account for greater than 90% of this burden [2].

The earliest reported Yellow Fever outbreak in Nigeria was in 1864 and between 1865 and 1996, the country continues to record outbreaks. A major outbreak reportedly occurred in April and May 1987 in Oyo State. The outbreak ended in July with 805 cases and 416 deaths officially reported. The principal vector was the *Aedes aegypti* breeding in domestic water containers [6]. After 21 years of no confirmed yellow fever cases, a resurgence of yellow fever outbreaks was documented [7] in 2017, with recorded confirmed cases within the Ifelodun Local Government Area (LGA) of Kwara State. This renewed onset of yellow fever outbreaks in Nigeria followed a global trend of reports from other African countries marking the emergence of yellow fever as a brand-new re-emerging global threat in 2016 [8]. Giant outbreaks of yellow fever were reported in Angola and the Democratic Republic of Congo which highlighted the gaps and challenges of surveillance, rapid diagnosis due to lack of Medical Laboratory capacity, and availability of global vaccine stockpiles resulting in poor routine vaccination coverage [8]. In addition, factors like porous borders and increased migration, the widespread distribution of *Aedes* mosquitoes, and lack of efficient health policies and surveillance systems, favour this complex epidemiological scenario of the reemergence of yellow fever [8].

The last decade shows a marked increase in the number of reported Yellow Fever outbreaks in the country with widespread viral transmission to many States between 2017 and 2019. In 2019 alone, 13 of the 36 States in Nigeria including Oyo State recorded at least one confirmed yellow fever case [3].

Currently, there is no cure for Yellow Fever disease, but treatment by management of presenting complaints, signs, and symptoms, and infection prevention and control through vaccination and vector control is the main method to prevent the spread of the virus [9]. There is the availability of safe and highly effective yellow fever vaccine usually given routinely to infants at 9 months and emergency vaccination of population at risk during outbreaks, as well as to travelers to high-risk areas to curtail the spread of the virus. A single dose of the vaccine provides effective immunity within 30 days of vaccination for 99% of the vaccine, conferring sustained immunity with life-long protection [1]. However, poor vaccination coverages continue to put a lot of the population at risk of the disease, especially in Africa.

Yellow Fever vaccination coverage in West African Countries under French rule was estimated to be over 80% as of 1960 with mandatory vaccination introduced by France, [10] susceptible population however continues to grow after the Independence of these countries. It is noteworthy that other countries in the region are not included in the mandatory vaccination. Estimations from a study conducted by Shearer et al. [11] in 2017 show increasing coverages since 1970, though with notable gaps within yellow fever risk zones, with about 472 million people still requiring yellow fever vaccination in high-risk areas. Results from the rapid yellow fever vaccination coverage assessment carried out in Kwara State Nigeria following the confirmed case in 2017 produce 46% yellow fever coverage in the LGAs. [7].

In 2017, World Health Organization developed the Eliminating Yellow fever Epidemic (EYE) strategy to improve high-risk countries’ prevention, preparedness, detection, management, and response to yellow fever outbreaks. This was launched in April 2018 and Nigeria is considered as one of the high-risk countries for yellow fever disease in Africa [12].

This paper describes the changing epidemiology of the yellow fever virus in Oyo State between 2013 and 2020, and examines the role of routine EPI coverage and quality of the surveillance activities in the transmission of yellow fever in Oyo state, after decades without an outbreak.

## Methodology

We conducted a retrospective cross-sectional study that examines the incidence and resurgence of Yellow fever outbreaks and immunization coverages from 2013 to 2020 in Oyo State, Nigeria.

### Study area

Oyo State is in the South-West geopolitical zone of Nigeria with Ibadan city as her capital. The State is located in the Southern Guinea Savannah region characterized by trees and grasses with the rainy season which lasts at least 7 months yearly. Oyo State has 33 Local Governments Areas (LGAs) and 29 Local Council Development Areas and a projected population of 9,233,010 with an annual growth rate of 3.2 [13]. The State is bounded to the North by Kwara State, to the East by Osun State, and Southwest by Ogun State and the Republic of Benin.

### Data extraction

 We reviewed the data for the state with a primary focus on surveillance and routine EPI coverage. Surveillance data were retrieved from the state’s database reported by all 33 LGAs, maintained by the State and supported by the World Health Organization at the Zonal and State levels. Immunization data were retrieved from District Health Information Software 2 (DHIS2) platform for the state.

The study made use of already collected data from the yellow fever case-based surveillance system between January 2013 and December 2020. In this surveillance system, the definition of suspected yellow fever is any person with acute onset of fever, with jaundice appearing within 14 days of onset of the first symptoms and a probable case is a suspected case that is epidemiologically linked to a confirmed case/ an outbreak or with positive post-mortem liver histopathology or both. Reported suspected cases within the IDSR were investigated. Case investigation form is used to collect information about such cases. Blood samples are collected from the suspected cases with cases information collected on the standardized laboratory form. Serum was separated from the blood samples and sent to the accredited National laboratory with appropriately filled forms for testing. ELISA IgM positives and inconclusive samples are sent to yellow fever reference laboratory Institute Pasteur in Dakar (IPD) for confirmatory testing.

The routinely collected immunization data on DHIS2 was retrieved for Oyo State and the vaccination status of all the suspected cases was assessed through the surveillance system database.

### Measurements

 We measured the proportion of LGAs reporting at least one case of suspected yellow fever with a blood specimen, and the number of suspected cases reported for each year within the period under review. We also assessed the trend of confirmed cases and the incidence per 100,000 persons. Also, suspected cases of yellow fever were categorized into four age groups and their routine EPI vaccination status was assessed.

 In addition, we also retrieved the data on annual administrative vaccination coverage for the yellow fever vaccine, juxtaposing it with the number of confirmed cases for each year during the period under review.

## Results

Table 1 shows Age Distribution of Suspected Yellow Fever and Surveillance Performance 2013-2020.

**Table 1:** Surveillance performance and age distribution of suspected yellow fever 2013-2020

Parameter	2013	2014	2015	2016	2017	2018	2019	2020
**Basic Indicators**								
Proportion of LGAs reporting at least 1 case with a blood specimen	15.2%	6.1%	33.3%	18.2%	39.4%	51.5%	84.9%	75.8%
Number of suspected YF Cases	6	2	20	8	19	40	94	51
Probable YF Cases	0	0	0	0	1	0	0	0
Confirmed YF Cases	0	0	0	0	0	0	3	5
Incidence per 100,000 population	0	0	0	0	0	0	3.5	5.6
**Age Groups**								
< 5 years	2	0	3	3	4	3	20	6
5 - 9 years	0	0	5	1	2	8	18	9
10 - 14 years	1	1	3	1	2	8	10	5
15 years +	3	1	9	3	11	21	46	31

The proportion of LGAs reporting at least a case of suspected yellow fever, with blood samples collected, ranged from 6.1 to 84.9% between 2014 and 2020. The findings further indicate a positive directional movement and an upward trend was observed from 2014 to 2019. The year 2020 however had reports of suspected yellow fever cases from 75.8% of LGAs in the State.

Overall, the lowest number, only 2 cases, of suspected yellow fever cases were recorded in 2014 while the highest, 94 suspected cases were recorded in 2019. A decline was observed in 2020 with only 51 suspected cases reported, though still considerably higher than other years.

In the period under review, a total of 9 confirmed (8cases) and probable (1 case) cases of yellow fever were recorded. However, there were no confirmed cases from the year 2013 to 2016, including 2018. One Probable case was recorded in the State in 2017 before the surge in 2019 to 2020, thus indicating an upward trend, which could also be observed in the upward trend of incidence of the disease per 100,000 population from 0% to 2013 through 2018, to 3.5% in 2019 and then to 5.6% in 2020.

The age group 15 years+ was observed to have the highest number of reported suspected Yellow Fever cases for each year from 2013 to 2020, with a total of 125 cases, accounting for 52% of all suspected cases reported. The descending order in the number of cases for the specified age groups was observed as 15 years+, 5 – 9 years, <5 years, with the age group 10 – 14 years being the least with 31 suspected cases reported.

Table 2 illustrates the Vaccination Status of suspected yellow fever cases from 2013 to 2020.

**Table 2:** Vaccination status of suspected yellow fever cases 2013-2020

Status	2013	2014	2015	2016	2017	2018	2019	2020	Cumulative	Proportion
Vaccinated	3	1	15	6	5	18	34	11	93	39%
Unvaccinated	3	1	5	2	14	5	12	19	61	25%
Unknown	0	0	0	0	0	17	48	21	86	36%
Proportion of vaccinated	50%	50%	75%	75%	26%	45%	36%	22%	39%	

The vaccination status was classified into 3 categories: vaccinated, unvaccinated and unknown.

**Fig. 1:**
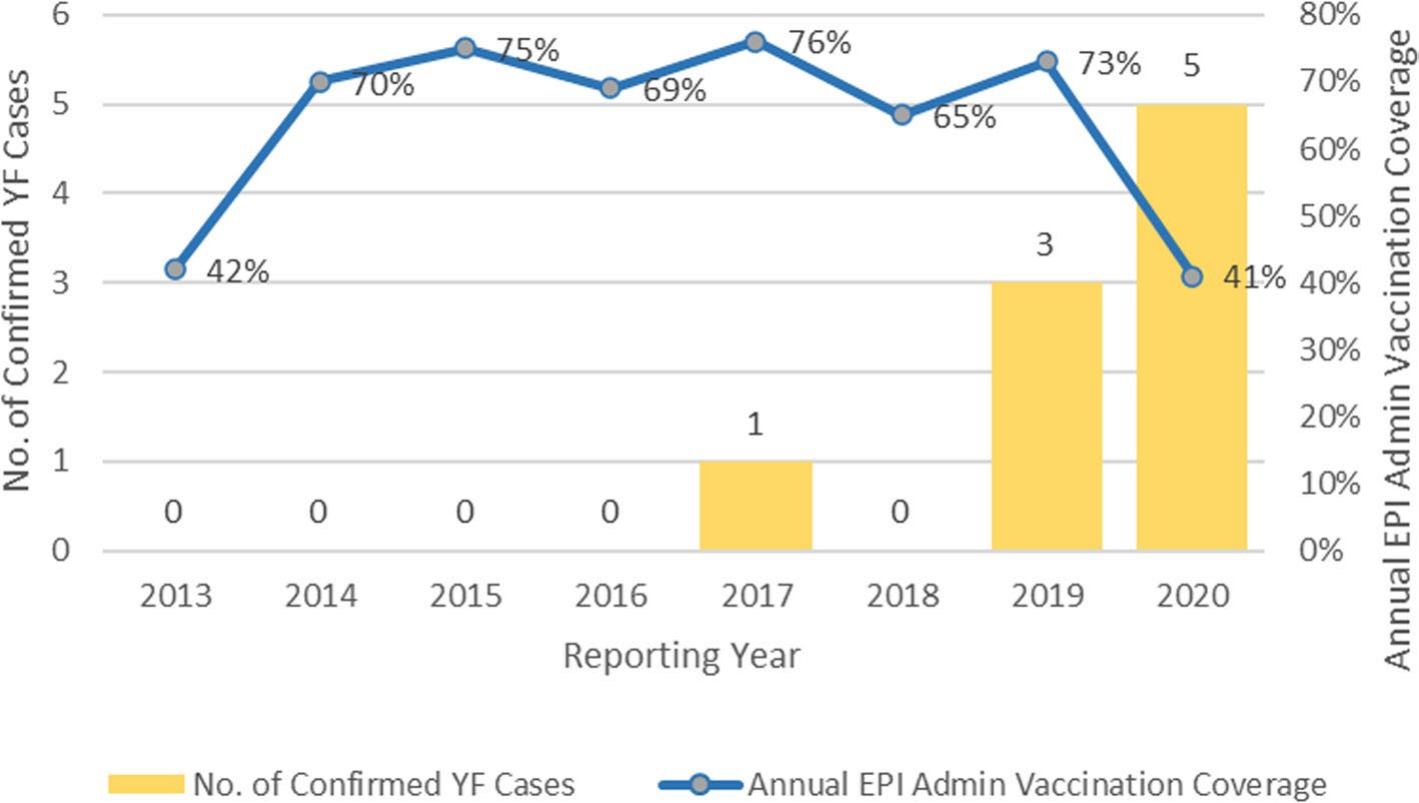
Indicates the number of confirmed yellow fever cases and the annual EPI administrative vaccination coverage 2013 to 2020

In total, 93 of 240 (39%) suspected yellow fever cases reported during the given period were observed to have received yellow fever vaccine. Furthermore, 61 of the 240 (25%) were unvaccinated, and 86 (36%) had unknown vaccination status. At least, half of the suspected yellow fever cases reported in the year 2013 to 2016 had received yellow fever vaccine while less than 50% of suspected cases in 2017 to 2020 had been vaccinated against Yellow Fever with the years 2017 and 2020 recording only about a quarter 26% and 22% respectively, of all suspected cases who had received Yellow Fever vaccine Fig. 1.

According to the data collected, a total of 8 confirmed and 1 probable case of yellow fever was recorded. The highest number of confirmed cases, 5 cases, was recorded in the same year, 2020 which happens to have the lowest (41%) annual EPI administrative coverage, followed closely by 2013 with 42% yellow fever annual routine immunization administrative coverage. Greater than 65% annual coverage was reported from 2014 to 2019 Fig. 2.

**Fig. 2:**
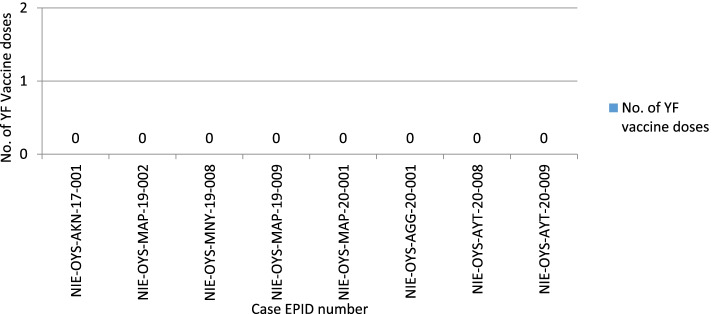
Indicates the vaccination status of confirmed yellow fever cases from 2013 to 2020

According to the data retrieved from reports of presumptive yellow fever outbreak investigations, none of the laboratory-confirmed cases had received a dose of the yellow fever vaccine.

## Discussion

In recent years, there has been an increase in the suspected and confirmed yellow fever cases in Oyo state. From 2016, there has been a gradual increase in the number of suspected cases and the proportion of LGAs reporting at least a suspected case with blood samples collected, which is an indication of an improved surveillance system. The highest number of suspected cases, 94, and the highest proportion of LGAs reporting, 84.9% were both recorded in 2019. The gradual increase could be linked with an increased capacity of surveillance officers, expansion of the surveillance network characterized by the inclusion of assistant disease surveillance and notification officers as well as an increase in the number of community informants at the LGA level, and provision of incentives to other components of the surveillance system. These interventions have increased the sensitivity of the surveillance system to detect cases of yellow fever in health facilities and the community. In addition, the visit of the African Regional Certification Committee (ARCC) in 2019 to the country and state led to the conduct of several key surveillance activities such as intensified active case search and intensified and extensive sensitization activities, which increased the sensitivity of the surveillance system.

After many years of no confirmed yellow fever case in Oyo State, a probable case was recorded in 2017 and this coincides with the same period for the same year of Nigeria’s first confirmed case in 21 years [14] which was reported in neighboring Kwara State. Then followed another 3 confirmed cases in the year 2019 and yet another 5 new cases in 2020 with an incidence rate per 100, 000 population rising from 0% to 2013 to 3.5% in 2019 and 5.6% in 2020. Though the year 2019 had 12 other States in Nigeria recording yellow fever outbreaks [3], most of which were linked to the Yankari game reserve in Bauchi State, the majority of the states are Northern parts of the country, nonetheless, Oyo State was one of the few Southern Nigeria States that recorded an outbreak. This can be linked to the re-emergence of the disease in the State and an indication of Oyo State being high risk for Yellow fever transmission.

In the period under review, the most affected age group was observed to be above 15 years. This could also be as a result of the level of exposure of individuals in this age group to the disease vector as observed in Delta and Enugu states. On the contrary, findings from the Kwara state outbreak in 2017 show that individuals below 15 years (55%) were mostly affected [14]. Children below the age of 10 years accounted for 35% of all reported suspected cases between 2013 and 2015 and this is similar to observation by Tomashek et al., 2019 that due to increasing natural immunity with age in Africa, infants and children are often most affected during yellow fever outbreaks. This is especially important because children can most likely be the major unprotected group in areas with poor yellow fever routine immunization coverage and especially the unprotected cohort where preventive or reactive mass yellow fever vaccination campaigns had previously been conducted.

Furthermore, it was observed that 3 out of the confirmed cases in 2020 were domiciled in agrarian communities. This is a major factor affecting the level of exposure to the disease vector and is in tandem with findings from the outbreak reported in Delta and Enugu state in 2020 [15] with a majority of the cases being farmers. In addition, it was observed that males were more affected than females. This could be directly linked to the farming occupation and by extension, a higher level of exposure of the males to the ecological niche of the disease vector. This is in sync with the 7894 cases reported in 20 states in the country from September 2017 to October 2019, with 57% being males. Findings from Brazil between 2017 and 2018 [16] also show that majority of those affected were males.

The vaccination status of all suspected cases between 2013 and 2020 was assessed and it was observed that only 39% of all suspected cases were vaccinated with at least 1 dose of yellow fever vaccine. This could be linked to the number of confirmed cases detected. Furthermore, with the WHO recommendation of 60–80% vaccination coverage to prevent outbreaks [9], a comparison of the annual EPI yellow fever vaccination coverages for each year in the period under review with the laboratory-confirmed cases was conducted in a bid to assess a link between both parameters. The finding revealed that the highest number of laboratory-confirmed cases were recorded in 2020, which was the year with the lowest vaccination coverage (41%). This is not surprising, as previous findings follow a similar pattern [7], and the yellow fever vaccine is said to be highly efficacious, conferring life-long immunity to 99% of persons vaccinated [9]. The low vaccination coverage and a high number of confirmed cases recorded in 2020 are largely linked to the disruption of service delivery, immunization, and health education on hygiene and vector control inclusive, due to the COVID-19 pandemic. The pandemic can thus be said to have contributed to outbreaks of other diseases including yellow fever in the country.

Greater than 65% annual yellow fever vaccination coverage was reported from 2014 to 2019, yet there was a record of yellow fever outbreaks in 2019. This may be because the vaccination data available for this study was that of the routine immunization data for the under 1/ under 2 years of age and so vaccination coverage for the whole population at risk may be less than the recommended 60-80% which can prevent the occurrence of outbreaks [5] and less than 80% that should prevent viral transmission [1]. This is a limitation of this work and future studies may incorporate data from the recently concluded mass yellow fever vaccination campaign and travelers’ yellow fever vaccination data.

In conclusion, the increase in the circulation of the yellow fever virus reiterates the state being at a high risk of yellow fever transmission and underlines the need for viable interventions such as environmental hygiene in a bid to rid the environment of the disease vector’s ecological niche and improving coverages for both routine EPI vaccination and mass vaccination campaigns to provide population immunity. Another alternative to improve immunization coverage may be to make the vaccine available at strategic locations at no cost to people above 2 years old who may have missed being vaccinated during mass vaccination campaigns. In addition, the introduction of a second dose of the yellow fever vaccine targeted to boost the immunity of individuals previously vaccinated could be essential in reducing the incidence of positive cases of yellow fever particularly in endemic areas, as there have been debates on seroconversion rates after yellow fever vaccination, waning immunity and primary vaccine failure [16].

## Data Availability

The data were generated as part of the activities supporting disease surveillance and routine immunization in Nigeria. The data are kept at the WHO server and are subject to protection. The datasets used for the current study are available from the corresponding author upon request.

## Abbreviations

ARCC: African Regional Certification Committee
EYE: Eliminating Yellow fever Epidemic
WHO: World Health Organization
(DHIS2): District Health Information Software 2
LGAs: Local Government Areas
RNA: Ribonucleic acid
IDSR: Integrated Disease Surveillance and Response
IPDs: Institute Pasteur in Dakar
COVID-19: Coronavirus Disease 2019
EPI: Expanded Programme on Immunization
